# Unveiling genetic variants for age-related sarcopenia by conducting a genome-wide association study on Korean cohorts

**DOI:** 10.1038/s41598-022-07567-9

**Published:** 2022-03-03

**Authors:** Heejin Jin, Hyun Ju Yoo, Ye An Kim, Ji Hyun Lee, Young Lee, Seung-hyun Kwon, Young Joo Seo, Seung Hun Lee, Jung-Min Koh, Yunmi Ji, Ah Ra Do, Sungho Won, Je Hyun Seo

**Affiliations:** 1 Institute of Health and Environment, Seoul National University, Seoul, South Korea; 2 Department of Convergence Medicine, Asan Institute for Life Sciences, Asan Medical Center, University of Ulsan College of Medicine, Seoul, South Korea; 3 Division of Endocrinology, Department of Internal Medicine, Veterans Health Service Medical Center, Seoul, South Korea; 4 Veterans Medical Research Institute, Veterans Health Service Medical Center, Jinhwangdo-ro 61-gil 53, Gangdong-gu, Seoul, 05368 South Korea; 5 Department of Anesthesiology and Pain Medicine, Hangang Sacred Heart Hospital, Hallym University College of Medicine, Seoul, South Korea; 6 Division of Endocrinology and Metabolism, Asan Medical Center, University of Ulsan College of Medicine, Seoul, South Korea; 7 Interdisciplinary Program of Bioinformatics, College of National Sciences, Seoul National University, Seoul, South Korea; 8 Department of Public Health Science, Institute of Health & Environment, Seoul National University, Seoul, South Korea; 9 RexSoft Corps, Seoul, South Korea

**Keywords:** Genetic association study, Geriatrics, Skeletal muscle, Genetics research

## Abstract

Sarcopenia is an age-related disorder characterised by a progressive decrease in skeletal muscle mass. As the genetic biomarkers for sarcopenia are not yet well characterised, this study aimed to investigate the genetic variations related to sarcopenia in a relatively aged cohort, using genome-wide association study (GWAS) meta-analyses of lean body mass (LBM) in 6961 subjects. Two Korean cohorts were analysed, and subgroup GWAS was conducted for appendicular skeletal muscle mass (ASM) and skeletal muscle index. The effects of significant single nucleotide polymorphisms (SNPs) on gene expression were also investigated using multiple expression quantitative trait loci datasets, differentially expressed gene analysis, and gene ontology analyses. Novel genetic biomarkers were identified for LBM (rs1187118; rs3768582) and ASM (rs6772958). Their related genes, including *RPS10*, *NUDT3*, *NCF2*, *SMG7,* and *ARPC5*, were differently expressed in skeletal muscle tissue, while *GPD1L* was not. Furthermore, the ‘*mRNA destabilisation*’ biological process was enriched for sarcopenia. Our study identified *RPS10*, *NUDT3,* and *GPD1L* as significant genetic biomarkers for sarcopenia. These genetic loci were related to lipid and energy metabolism, suggesting that genes involved in metabolic dysregulation may lead to the pathogenesis of age-related sarcopenia.

## Introduction

Sarcopenia is the age-related loss of skeletal muscle mass and strength, accompanied by functional impairment. As such, it is associated with disability, poor quality of life, and increased mortality^1–3^. Considering the difficulties posed by frailty, and the healthcare costs associated with age-related conditions, such as sarcopenia^4,5^, it is necessary to identify meaningful disease phenotypes and biomarkers. Several studies have suggested various criteria for defining sarcopenia^6–10^. Muscle mass is thought to be an important factor for the diagnosis of sarcopenia; of the parameters related to muscle mass, lean body mass (LBM) is frequently used to predict sarcopenia. In addition, the Asian working group for sarcopenia 2019 (AWGS 2019) recently reached the consensus that skeletal muscle index (SMI) and appendicular skeletal muscle (ASM) may also be reliable parameters^11^. In this respect, in addition to LBM, it is necessary to analyse SMI and ASM to understand the complex aetiology of sarcopenia, which can be attributed to a variety of factors, including oxidative stress, inflammation, mitochondrial dysregulation, and genetic factors^12,13^.

Muscle mass has a genetic trait phenotype, with a heritability estimate of over 50%^14^. Studies have investigated the genetic factors of LBM using the associations of single nucleotide polymorphisms (SNPs)^15–19^. Moreover, as osteoporosis and sarcopenia share a common risk factor (ageing), several studies have conducted joint genome-wide association study (GWAS) analyses on overlapping genetic variants^20,21^. Notably, a GWAS into osteoporosis revealed 64 loci. In contrast, fewer loci were identified by a GWAS into muscle-related phenotypes, thus. providing fewer biological insights into pathways regarding sarcopenia^20^. To address this issue, a large GWAS meta-analysis was conducted using 20 cohorts of European ancestry, identifying a set of five loci (*HSD17B11*, *VCAN*, *ADAMTSL3*, *IRS1* and *FTO*) for total LBM, and SNPs related to *IRS1*, *ADAMTSL3,* and *VCAN* for appendicular LBM^22^. However, as this study analysed the entire cohort, irrespective of age, its findings regarding genes associated with sarcopenia as a senile disease were limited. In addition, the GWAS was based on European ancestry, and little is known regarding genetic determinants in elderly East Asians. Still further, few genetic studies have utilised a new index for sarcopenia (released by the AWGS in 2019) to investigate ASM or SMI^11^. Thus, a need exists for the investigation of genetic components associated with sarcopenia using multiple cohorts comprising elderly East Asians. The current study conducted a GWAS meta-analysis on sarcopenia phenotypes using Korean relatively aged cohorts, combining the Veterans Health Service Medical Center (VHSMC) and Korean Association Resource (KARE) cohorts.

## Results

### Characteristics of the study participants

**Figure 1:**
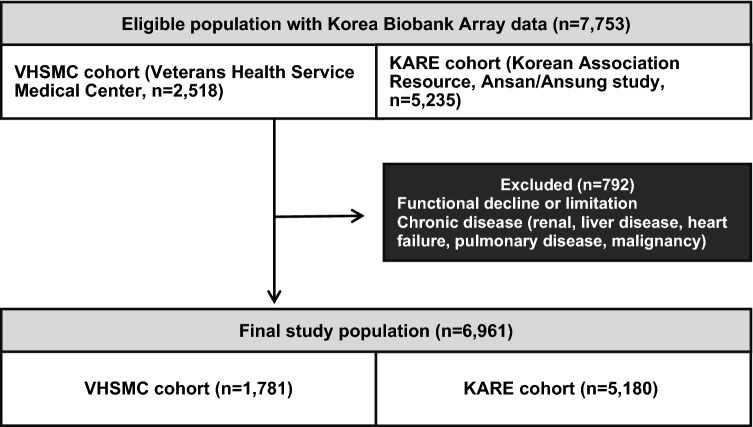
Schematic of study population.

A total of 7753 eligible subjects were included in this study (2518 subjects from the VHSMC cohort and 5235 from the KARE cohorts). However, 792 were excluded due to the exclusion criteria (Fig. 1), leaving a remainder of 6961 participants (1781 subjects from the VHSMC cohort and 5180 from the KARE cohort) that were included in analyses. The mean age of the VHSMC cohort was higher than that of the KARE cohort (69.10 ± 7.83 years vs 62.79 ± 8.33 years, *P* < 0.001, Table 1). No significant difference was observed in mean height (1.59 ± 0.08 m in VHSMC vs 1.59 ± 0.09 m in KARE, *P* = 1.000) between the two cohorts. The mean weight (63.24 ± 10.51 kg in VHSMC vs 62.64 ± 10.37 kg in KARE, *P* = 0.037) and BMI (24.74 ± 3.21 kg/m^2^ in VHSMC vs 24.53 ± 3.15 kg/m^2^ in KARE, *P* = 0.016) were statistically different between the cohorts. The LBM of the VHSMC cohort was lower than that of the KARE cohort (40.10 ± 7.83 kg vs 42.03 ± 8.27 kg, respectively, *P* < 0.001), whereas the body fat mass (BFM) of the VHSMC cohort was higher than that of the KARE cohort (20.60 ± 6.23 kg vs 18.28 ± 5.85 kg, respectively, *P* < 0.001). Descriptive statistics for subgroups according to sex are also presented in Table 1. The mean values of SMI and ASM, which could only be calculated for the VHSMC cohort, were 6.77 ± 1.00 kg/m^2^ and 17.49 ± 4.06 kg, respectively.

**Table 1:** Baseline characteristics of study populations.

Cohort	Entire			Male			Female		
	VHSMC	KARE	*P*	VHSMC	KARE	*P*	VHSMC	KARE	*P*
Numbers	1781	5180		712	2449		1069	2731	
Sex (male)	712 (40%)	2449 (47%)	0.001^a^	N/A	N/A	N/A	N/A	N/A	N/A
Age (years)	69.10 ± 7.83	62.79 ± 8.33	< 0.001^b^	71.11 ± 7.34	62.20 ± 8.03	< 0.001^b^	67.77 ± 7.86	63.32 ± 8.56	< 0.001^b^
Height (m)	1.59 ± 0.08	1.59 ± 0.09	1.000^b^	1.67 ± 0.05	1.66 ± 0.06	< 0.001^b^	1.55 ± 0.05	1.53 ± 0.06	< 0.001^b^
Weight (kg)	63.24 ± 10.51	62.64 ± 10.37	0.037^b^	69.95 ± 9.48	67.79 ± 9.68	< 0.001^b^	58.77 ± 8.62	58.00 ± 8.63	0.013^b^
BMI (kg/m^2^)	24.74 ± 3.21	24.53 ± 3.15	0.016^b^	24.99 ± 3.02	24.32 ± 2.94	< 0.001^b^	24.57 ± 3.32	24.72 ± 3.33	0.211^b^
LBM (kg)	40.24 ± 7.60	42.03 ± 8.27	< 0.001^b^	47.69 ± 6.23	48.86 ± 6.01	< 0.001^b^	35.90 ± 4.21	35.90 ± 4.21	< 0.001^b^
BFM (kg)	20.60 ± 6.23	18.28 ± 5.85	< 0.001^b^	20.60 ± 6.23	16.32 ± 5.32	< 0.001^b^	20.04 ± 5.75	20.04 ± 5.75	< 0.001^b^
SMI (kg/m^2^)	6.77 ± 1.00	N/A	N/A	7.67 ± 0.72	N/A	N/A	6.18 ± 0.65	N/A	N/A
ASM (kg)	17.49 ± 4.06	N/A	N/A	21.50 ± 2.80	N/A	N/A	14.82 ± 2.10	N/A	N/A

ASM, appendicular skeletal muscle; BMI, body mass index; LBM, lean body mass; BFM, body fat mass; SMI, skeletal muscle mass index; VHSMC, Veterans Health Service Medical Center; KARE, Korean Association Resource; N/A, not available.

^a^Chi-square test, ^b^t-test.

### GWAS meta-analysis of lean body mass and body fat mass

**Figure 2:**
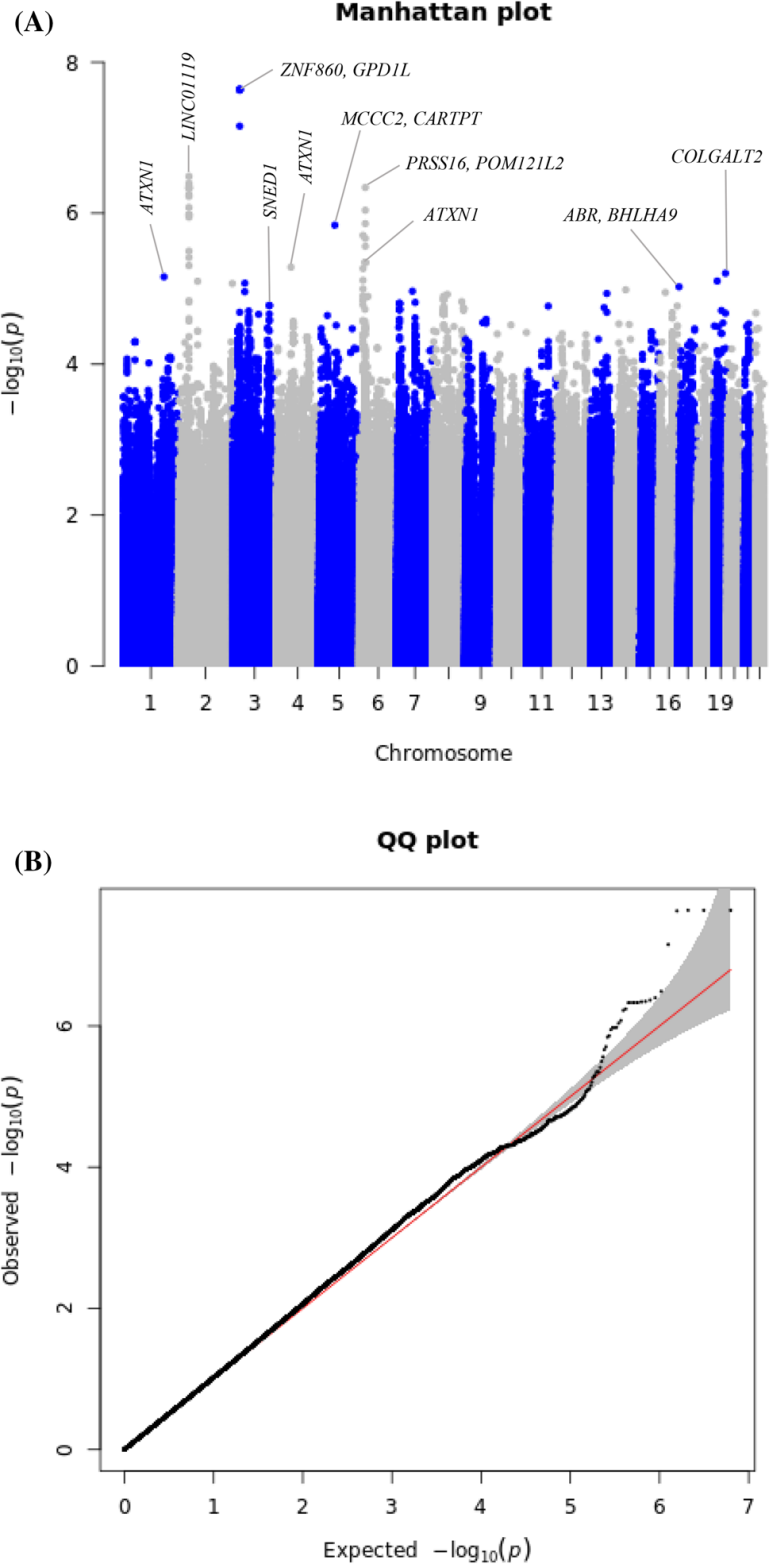
Manhattan and quantile–quantile plot for lean body mass in the meta-analysis. (**A**) Manhattan plot of the *P*-values in the genome-wide association study (GWAS) meta-analysis for lean body mass. (**B**) Quantile–quantile (Q-Q) plot showing expected vs. observed [− log_10_(*P*)values]. The expected line is shown in red and confidence bands are shown in grey.

A total of 2,360,975 SNPs were used for the GWAS meta-analysis of LBM and BFM. Quantile–quantile (Q-Q) and Manhattan plots for LBM are shown in Fig. 2. The Q-Q plot revealed no evidence of test statistic inflation (variance inflation factor [VIF] = 1.044). The top ten variants for LBM are listed in Table 2; two of which were genome-wide significant loci. The most significant variant was rs1187118 (effect = 0.720, standard error [SE] = 0.117, *P* = 1.09 × ${10}^{-9},$ *HetPVal* = 0.199) near *Glutamate Metabotropic Receptor 4* (*GRM4*) and *High Mobility Group AT-Hook 1* (*HMGA1*), followed by rs3768582 (effect = 0.554, SE = 0.100, *P* = 4.09 ×  ${10}^{-8}$, *HetPVal* = 0.537) near *Neutrophil Cytosolic Factor 2* (*NCF2*). The remaining eight variants are presented as candidate loci in Table 2. The Q-Q and Manhattan plots for BFM are shown in Supplementary Fig. S1. The Q-Q plot revealed no evidence of test statistic inflation (VIF = 1.037). The GWAS meta-analysis for BFM showed no genome-wide significant loci and the variant with the smallest *P*-value was rs1592269 (effect = 0.753, SE = 0.148, *P* = 3.43 ×  ${10}^{-7}$, *HetPVal* = 0.379) near *GRM4* and *HMGA1*. The top ten candidate loci associated with BFM with *P*-values < 1.00 × ${10}^{-5}$ are listed in Supplementary Table S1. As the GWAS results for the LBM and BFM phenotypes exhibited similar loci (*GRM4* and *HMGA1*), linkage disequilibrium (LD) analysis was performed. A high (r^2^ = 0.935) LD between rs1187118 and rs1592269 was observed, indicating a relatively high dependency.

**Table 2:** Results of GWAS meta-analysis for lean body mass (leading SNPs, top 10).

Chr	SNP	Position	Allele	MAF	Independent study					Meta-analysis			Mapped genes
					Cohort	Effect	SE	MAF	*P*	Effect	SE	*P (*HetPVal)	
6	rs1187118	34169020	A/T	0.16^a^, 0.11^b^	VHSMC	0.488	0.208	0.16	$1.89\times {10}^{-2}$	0.720	0.117	$1.09\times {10}^{-9}$ (0.1995)	*GRM4, HMGA1* (intergenic)
					KARE	0.831	0.143	0.17	$7.92\times {10}^{-9}$				
1	rs3768582	183553870	C/T	0.27^a^, 0.35^b^	VHSMC	0.637	0.177	0.23	$3.42\times {10}^{-4}$	0.554	0.100	$4.09\times {10}^{-8}$ (0.5376)	*NCF2* (intronic)
					KARE	0.516	0.122	0.27	$2.63\times {10}^{-5}$				
15	rs12441313	59015488	G/T	0.33^a^, 0.35^b^	VHSMC	0.473	0.161	0.32	$3.35\times {10}^{-2}$	0.475	0.094	$4.74\times {10}^{-7}$ (0.8894)	*ADAM10* (intronic)
					KARE	0.477	0.116	0.33	$4.19\times {10}^{-5}$				
22	rs4822064	42279506	G/C	0.05^a^, 0.06^b^	VHSMC	0.663	0.350	0.05	$5.91\times {10}^{-2}$	0.974	0.199	$1.05\times {10}^{-6}$ (0.3081)	*SREBF2* (intronic)
					KARE	1.124	0.243	0.05	$3.89\times {10}^{-6}$				
6	rs7453329	58721,863	C/T	0.41^a^, 0.37^b^	VHSMC	0.436	0.154	0.41	$4.81\times {10}^{-3}$	0.426	0.090	$2.39\times {10}^{-6}$ (0.8283)	*LINC00680-GUSBP4, NONE* (intergenic)
					KARE	0.421	0.111	0.41	$1.51\times {10}^{-4}$				
7	rs77502935	157290819	C/T	0.11^a^, 0.10^b^	VHSMC	0.430	0.241	0.12	$7.50\times {10}^{-2}$	0.651	0.138	$2.56\times {10}^{-6}$ (0.3043)	*LOC101927914* (ncRNA_intronic)
					KARE	0.762	0.170	0.11	$7.71\times {10}^{-6}$				
8	rs12156192	40763707	C/A	0.05^a^, 0.04^b^	VHSMC	− 0.663	0.300	0.07	$2.75\times {10}^{-2}$	− 0.805	0.174	$3.83\times {10}^{-6 }$(0.6457)	*ZMAT4,SFRP1* (intergenic)
					KARE	− 0.879	0.215	0.06	$4.37\times {10}^{-5}$				
3	rs12497693	72400695	C/T	0.40^a^, 0.34^b^	VHSMC	0.624	0.155	0.39	$6.15\times {10}^{-5}$	0.414	0.089	$4.33\times {10}^{-6}$ (0.0831)	*LINC00870, RYBP* (intergenic)
					KARE	0.311	0.109	0.40	$4.52\times {10}^{-3}$				
3	rs73872711	141110287	C/G	0.28^a^, 0.27^b^	VHSMC	0.401	0.166	0.30	$1.61\times {10}^{-2}$	0.437	0.096	$6.11\times {10}^{-6}$ (0.8804)	*ZBTB38* (intronic)
					KARE	0.457	0.119	0.29	$1.26\times {10}^{-4}$				
11	rs111357357	3640972	C/T	0.16^a^, 0.13^b^	VHSMC	0.421	0.208	0.16	$4.30\times {10}^{-2}$	0.533	0.119	$8.29\times {10}^{-6}$ (0.5698)	*LOC115995503, TRPC2* (intergenic)
					KARE	0.589	0.146	0.17	$6.00\times {10}^{-5}$				

Chr, chromosome; SNP, single nucleotide polymorphism; MAF, minor allele frequency; SE, standard error; Mapped Genes from ANNOVAR; GWAS, genome-wide association study; VHSMC, Veterans Health Service Medical Center; KARE, Korean Association Resource.

^a^Kref, Korean reference data; ^b^GnomAD Genome Aggregation Database (east Asian).

### GWAS of appendicular skeletal muscle and skeletal muscle index

**Figure 3:**
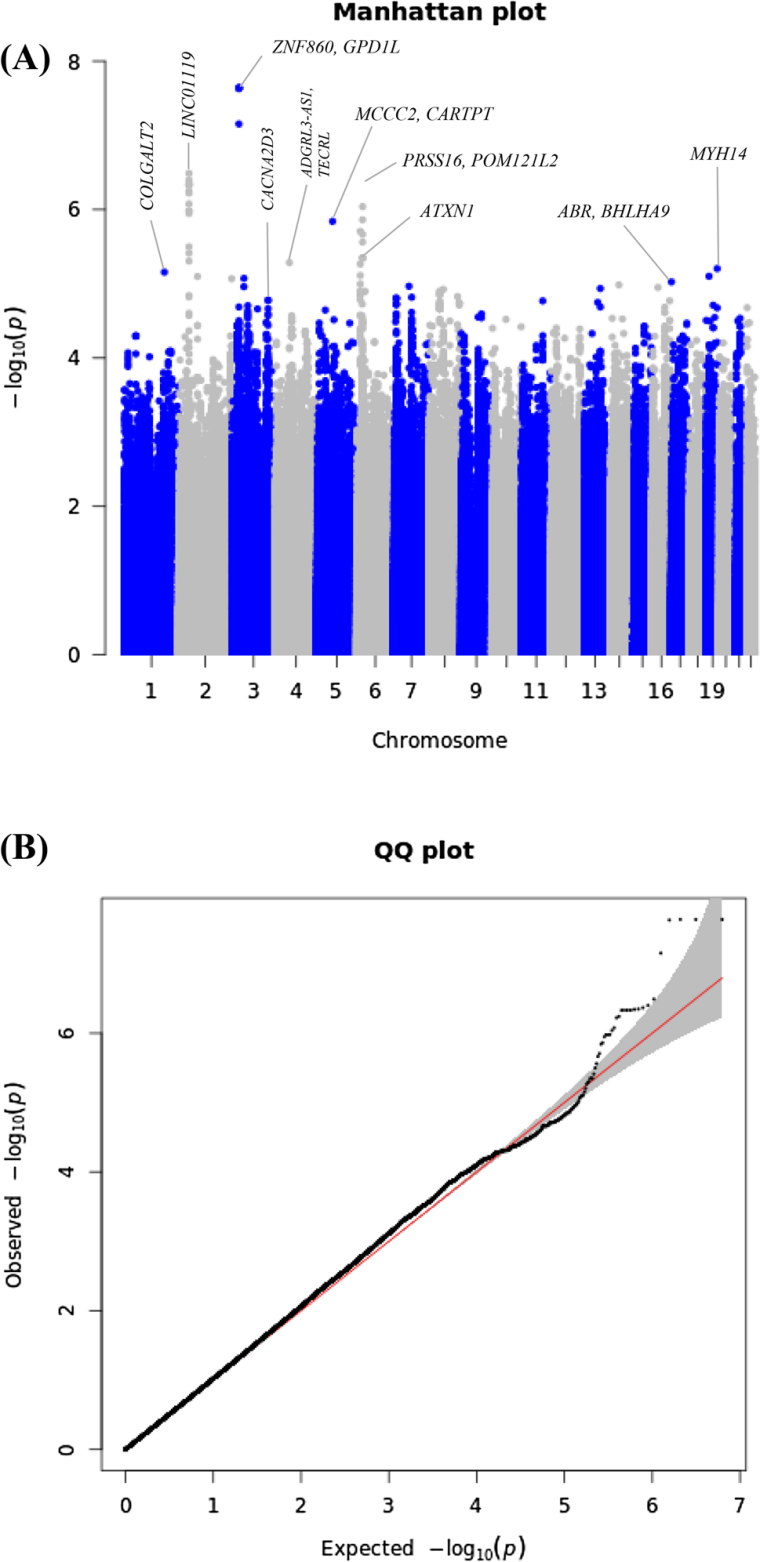
Manhattan and quantile–quantile plot for appendicular skeletal muscle in the genome-wide association analysis. (**A**) Manhattan plot of the *P*-values in the genome-wide association study (GWAS) meta-analysis for appendicular skeletal muscle. (**B**) Quantile–quantile (Q-Q) plot showing expected vs. observed [− log_10_(*P*)values]. The expected line is shown in red and confidence bands are shown in grey.

A total of 2,804,834 SNPs were used for GWAS analyses of ASM and SMI, using only the VHSMC cohort. The Q-Q and Manhattan plots for ASM are shown in Fig. 3; the Q-Q plot did not exhibit evidence of test statistic inflation (VIF = 1.031). The top ten variants for ASM are listed in Table 3; the only significant variant was a genome-wide locus: rs6772958 (effect = − 0.456, SE = 0.081, *P* = 2.30 ×  ${10}^{-8}$) near *zinc finger protein 860* (*ZNF860*) and *Glycerol-3-Phosphate Dehydrogenase 1 Like* (*GPD1L*). The Q-Q and Manhattan plots for SMI are shown in Supplementary Fig. S2 and revealed no evidence of test statistic inflation (VIF = 1.034). However, the GWAS for SMI exhibited genome-wide significant loci; the variant with the smallest *P*-value was rs6772958 (effect = − 0.121, SE = 0.023, *P* = 1.72 ×  ${10}^{-7}$, *HetPVal* = 0.379) near *ZNF860* and *GPD1L*. The top ten candidate loci with *P*-values < 1.00 × ${10}^{-5}$ are suggested in Supplementary Table S2.

**Table 3:** Results of GWAS for appendicular skeletal muscle (leading SNPs, top 10).

Chr	SNP	Position	Allele	MAF	Effect	SE	*P*	Mapped genes
3	rs6772958	32049091	A/G	0.39^a^, 0.39^b^, 0.45^c^	− 0.456	0.081	$2.30\times {10}^{-8}$	*ZNF860, GPD1L* (intergenic)
2	rs1466024	47065406	T/C	0.38^a^, 0.37^b^, 0.37^c^	0.430	0.008	$3.24\times {10}^{-7}$	*LINC01119* (ncRNA_intronic)
6	rs4713078	27227782	T/G	0.10^a^, 0.11^b^, 0.07^c^	0.728	0.147	$9.13\times {10}^{-7}$	*PRSS16, POM121L2* (intergenic)
5	rs16870827	70976503	A/G	0.13^a^, 0.11^b^, 0.22^c^	− 0.567	0.117	$1.44\times {10}^{-6}$	*MCCC2, CARTPT* (intergenic)
6	rs2146753	16694501	A/G	0.34^a^, N/A, 0.34^c^	− 0.401	0.084	$1.97\times {10}^{-6}$	*ATXN1* (intronic)
4	rs142722457	64547838	A/G	0.06^a^, 0.06^b^, 0.04^c^	− 0.792	0.173	$5.19\times {10}^{-6}$	*ADGRL3-AS1, TECRL* (intergenic)
19	rs1947424	50750497	C/T	0.15^a^, 0.15^b^, 0.17^c^	− 0.481	0.106	$6.24\times {10}^{-6}$	*MYH14* (intronic)
1	rs12089250	183983087	C/T	0.37^a^, 0.38^b^, 0.43^c^	0.375	0.083	$6.97\times {10}^{-6}$	*COLGALT2* (intronic)
3	rs11130441	55008440	C/T	0.42^a^, 0.42^b^, 0.42^c^	− 0.360	0.080	$8.46\times {10}^{-6}$	*CACNA2D3* (intronic)
17	rs59567306	1143024	A/G	0.06^a^, 0.08^b^, 0.12^c^	− 0.744	0.167	$9.44\times {10}^{-6}$	*ABR, BHLHA9* (intergenic)

Chr, chromosome; SNP, single nucleotide polymorphism; MAF, minor allele frequency; SE, standard error; Mapped Genes from ANNOVAR; GWAS, genome-wide association study.

^a^VHSMC, Veterans Health Service Medical Center; ^b^Kref, Korean reference data; ^c^GnomAD, Genome Aggregation Database (east Asian).

### Regional analysis and functional annotation

**Figure 4:**
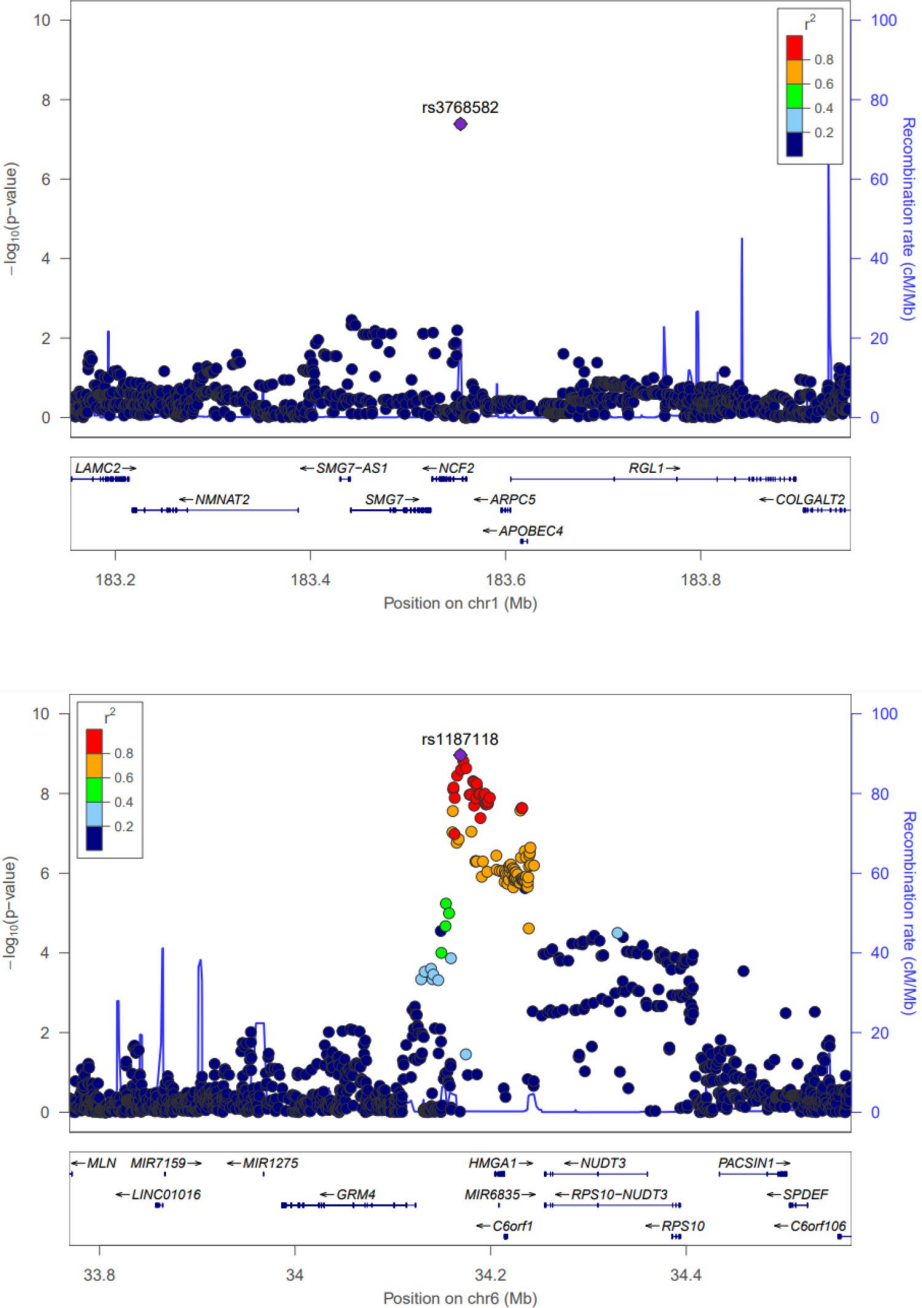
LocusZoom plot of genome-wide significantly associated SNPs for lean body mass.

**Figure 5:**
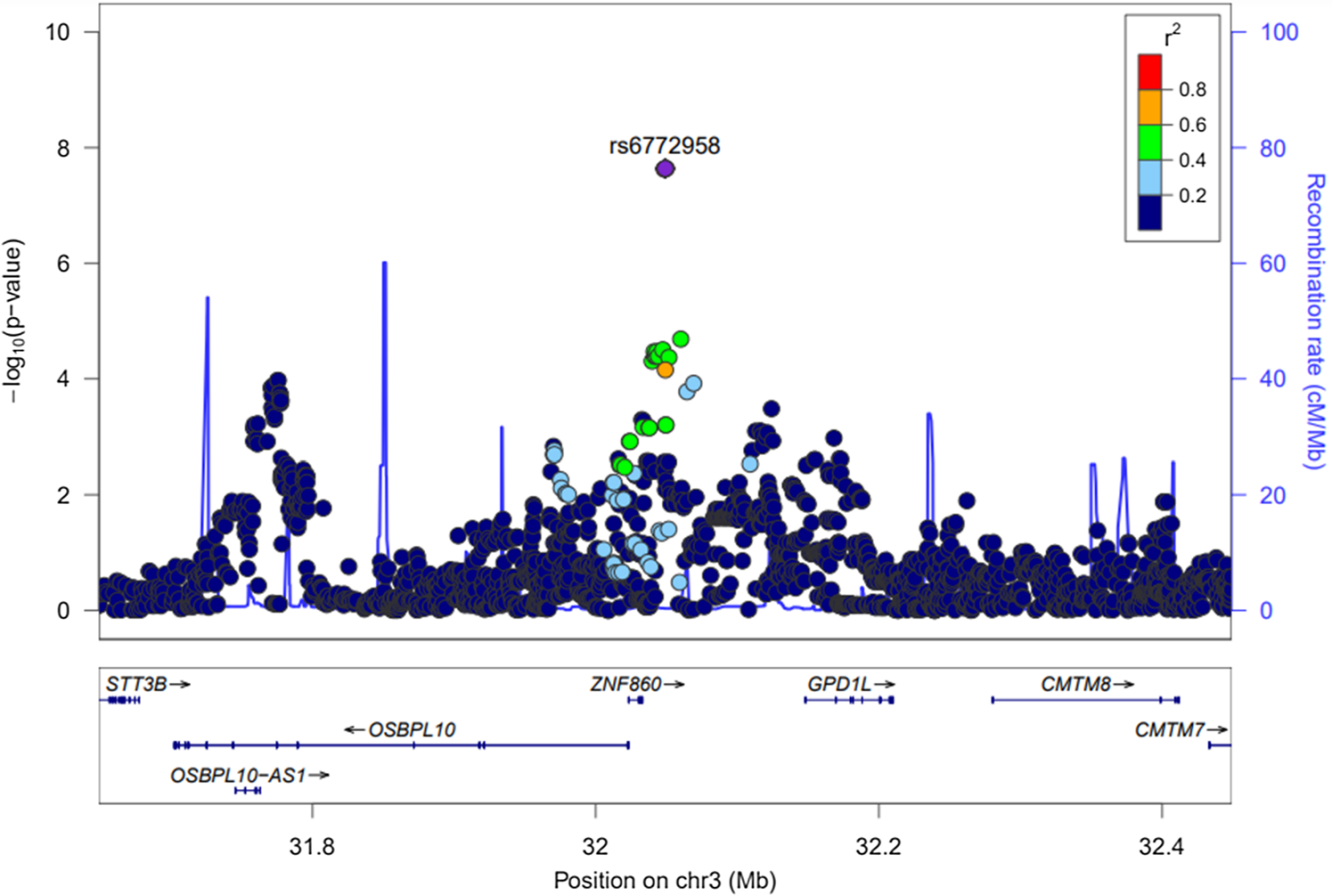
LocusZoom plot of genome-wide significantly associated SNPs for appendicular skeletal muscle.

For the genome-wide significant variants of each phenotype (LBM and ASM), the regional plots with the lead SNPs are displayed in Figs. 4 and 5. The first phenotype of interest was LBM. The most genome-wide significant SNP, rs1187118 eQTL analyses from the GTEx Project (V7), showed that *Ribosomal Protein S10* (*RPS10*) was highly expressed in skin sun-exposed lower leg tissue (*P* = 1.40 ×  ${10}^{-7}$). Its LD variant eQTL association for *Nudix Hydrolase 3* (*NUDT3*) was also found in the skeletal muscle tissue (*P* = 4.30 × ${10}^{-21}$). The second genome-wide significant SNP, rs3768582 eQTL analyses, showed that *NCF2*, *SMG7* (*SMG7 Nonsense-Mediated mRNA Decay Factor*) and *Actin Related Protein 2/3 Complex Subunit 5* (*ARPC5*) were highly expressed in the artery (*P* = 1.50 ×  ${10}^{-9}$), heart (*P* = 2.60 ×  ${10}^{-5}$), and cultured fibroblast tissue (*P* = 7.30 ×  ${10}^{-5}$), respectively. In the differently expressed gene (DEG) analysis with GSE38718, compared with the young group, *RPS10* (*P* = 8.00 ×  ${10}^{-4}$), *NUDT3* (*P* = 1.19 ×  ${10}^{-3}$), *NCF2* (*P* = 1.26 ×  ${10}^{-2}$), *SMG7* (*P* = 1.03 ×  ${10}^{-3}$) and *ARPC5* (*P* = 4.26 ×  ${10}^{-2}$) were more expressed in the elderly group (Table 4).

**Table 4:** Results of differentially expressed genes from Gene Expression Omnibus (GEO) databases.

Data set	Platform	Sample size	Gene	log FC	AveExpr	*P*-value	B
GSE38718	GPL570	Total: 22 Young: 14 Old: 8 Biceps brachii muscle	*RPS10*	− 0.752	5.026	8.00 × ${10}^{-4}$	− 0.556
			*NUDT3*	− 0.528	7.284	1.19 × ${10}^{-3}$	− 0.904
			*NCF2*	− 0.774	4.457	1.26 × ${10}^{-2}$	− 3.074
			*SMG7*	− 0.609	5.886	1.03 × ${10}^{-3}$	− 0.766
			*ARPC5*	− 0.185	4.672	4.26 × ${10}^{-2}$	− 4.148
			*GPD1L*	− 0.259	10.513	0.159	− 5.238

logFC = estimate of the log2-fold-change corresponding to the effect or contrast; AveExpr = average log2-expression for the probe over all arrays and channels; B = log-odds that the gene is differentially expressed.

The second phenotype of interest was ASM. The only genome-wide significant SNP, rs6772958 eQTL analysis, showed that *GPD1L* was highly expressed in thyroid tissue (*P* = 8.10 ×  ${10}^{-15}$). However, the DEG analysis showed that *GPD1L* expression was not significant (*P* = 0.159) in the transcriptome study (GSE38718).

In addition, gene ontology (GO) analyses of biological processes revealed that the term ‘*mRNA destabilisation (GO: 0061157)*’ (FDR-adjusted *P* = 0.090) was enriched, which is involved in skeletal muscles related genes. The term contains a pathway of alpha-ketoglutarate-dependent dioxygenase FTO (U6 small nuclear RNA [2′-O-methyladenosine-N(6)-]-demethylase FTO), which is involved in the regulation of fat mass, adipogenesis, and body weight. Thus, it contributes to the regulation of body size and body fat accumulation^23^.

## Discussion

This study discovered novel genetic biomarkers of LBM (rs1187118) and ASM (rs6772958) from the VHSMC and KARE cohorts, which comprise relatively aged (mean age: 69.10 vs. 62.79, respectively) Koreans. Their related genes for LBM, such as *RPS10, NUDT3, NCF2, SMG7,* and *ARPC5*, were expressed in skeletal muscle tissue. In addition, in the biological process, the term ‘*mRNA destabilisation* (*GO: 0061157*)’ (FDR-adjusted *P* = 0.090) was enriched for sarcopenia. This process contains alpha-ketoglutarate-dependent dioxygenase FTO. These results suggest that the pathogenesis of sarcopenia requires further investigation using a metabolic pathway linked to *mRNA*.

The aetiology of sarcopenia is complex and includes oxidative stress, inflammation, inadequate diets, a sedentary lifestyle, and genetic factors^13^. A previous study on genetic markers for sarcopenia identified the loci near *FTO*, *ESR1*, *NOS3*, *KLF5,* and *HLA-DQA1* to be associated with physical phenotypes, such as low handgrip strength and decreased LBM^24–26^. Nonetheless, these identified loci can only explain a small portion of phenotypic variations; thus, additional genetic loci should be identified. A recent large meta-analysis of the Cohorts for Heart and Ageing Research in Genome Epidemiology (CHARGE) Consortium and various other cohorts identified only a few loci, such as *FTO* and *VCAN* for LBM^22^. Therefore, assuming that identifying genetic variants for sarcopenia is challenging, we conducted GWAS analysis on a cohort comprising elderly subjects. The findings revealed that several genetic variants related to metabolism could be of importance in determining the pathogenesis of sarcopenia. Previous sarcopenia GWAS for European descendants showed association with *FTO*^22,27,28^ and several loci, including *TGFA* and *HLA-DRB1*^29^.

Our meta-analysis for both LBM and BFM showed significant differences in the intergenic area of *GRM4* and *HMGA1*, with a high LD between rs1187118 and rs1592269. *HMGA1* is overexpressed in adipose tissue, impairs adipogenesis, and prevents diet-induced obesity, and insulin resistance^30^. The top loci for LBM and BFM were similar, and those of ASM and SMI were similar since the parameter of LBM was calculated from body weight minus BFM, and SMI was calculated from ASM/height^2^. Hence, it would be useful to calculate the correlation and genetic correlation for each parameter. In VHSMC cohorts, the correlation and genetic correlation were 0.078 and 0.078 between LBM and BFM, respectively, whereas those between SMI and ASM were 0.948 and 0.948, respectively. In KARE cohorts, the correlation was − 0.02 between LBM and BFM with the genetic correlation being 0.349.

The eQTL analysis for muscle mass using GTEx datasets showed that *RPS10*, *NUDT3*, *NCF2*, *SMG7,* and *ARPC5* were differentially expressed in the muscle tissue for sarcopenia. However, this finding requires further validation. As the regional locations of *HMGA1*, *RPS10,* and *SIMM29* were in the upper stream of *NUDT3*, and may represent a regulatory function for the association of *NUDT3* with sarcopenia, further focus should be directed towards *NUDT3*. A previous study by Singh et al*.* suggested that *NUDT3* was a candidate target-locus, and emphasised the need for real-world validation using transcriptome-wide association study (TWAS) approaches that combine GWAS and eQTL summary data^24^. In the current study, *NUDT3* was found to be related to LBM in an elderly cohort. *NUDT3* belongs to the *MutT* or *Nudix* protein families, which act as homeostatic checkpoints at important stages in inositol phosphate metabolic pathways. These pathways, such as phosphatidyl-1d-myo-inositol and glycerophospholipid metabolism, from the Kyoto Encyclopedia of Genes and Genomes (KEGG) pathway database (https://www.kegg.jp/pathway/map00564)^31^ may, therefore, be related to LBM. For these reasons, it is necessary to understand the metabolic aspects of sarcopenia. A study into DEGs in skeletal muscle tissues from patients with cachexia^32^ showed that *NCF2* was identified from signal pathways related to inflammation. These findings are consistent with the findings of the present study. *SMG7* encodes a protein that is essential for nonsense-mediated *mRNA* decay, which is related to body height and BMI-adjusted waist circumference from a GWAS catalogue (https://www.ebi.ac.uk/gwas/). As SMG7 is linked with telomerase reverse transcriptase (TERT), sarcopenia may be related to muscle cell senescence via microRNA-195^33^. In addition, *ARPC5* encodes the actin related protein2/3 complex, which exhibited a negative fold change in expression related to the cytoskeleton in muscle tissue^34^. As these findings may represent secondary changes, or may be postulated from bioinformatics analysis, further studies are needed.

Additionally, the present study found that *GPD1L* is a significant genetic marker for ASM and SMI in the VHSMC cohort. Although this is a novel finding for GWAS using ASM as a parameter for sarcopenia, it requires further validation by studies from several cohorts. A tissue-based study into rat muscle identified *GPD1L* as a candidate locus for sarcopenia^35^. These findings were also observed in a previous study that investigated the sarcopenic muscle tissue of elderly women^36^, in which *GPD1L* was found to be downregulated via cytoplasmic energy metabolism. In addition, a systemic genetic approach identified that *GPD1L* and its molecular mechanism for obesity in human adipose tissue were associated with energy metabolism^37^. *GPD1L* expression was found to be negatively correlated with microRNA-210 (miR-210) levels, and was consistently downregulated in obese subjects^37^. They hypothesised that the decreased miR-210 levels increased *GPD1L*, thus inhibiting hypoxic transcription factor-1α (HIF-1α) activity. A previous study into the circulating miRNAs in plasma revealed that miR-210 is significantly downregulated in elderly patients with sarcopenia, compared to patients without sarcopenia^38^. Combined with results of previous studies^37,38^, the findings presented here suggest that *GPD1L* could be a genetic biomarker for sarcopenia, based on both miR-210 and HIF-1α pathways. Hence, an additional biomarker for sarcopenia may be postulated from this metabolic research. Recent studies into plasma biomarkers for sarcopenia have identified higher levels of amino acids and lower levels of phosphatidylcholines (PCs) and lysophosphatidylcholine (lysoPC)^39,40^. The association between *GPD1L* and PCs or lysoPC and sarcopenia may involve (1) dysregulation of *GPD1L* related to decreased PCs and lysoPC from previous lipid biomarkers^39,40^, or (2) an increase in the glycerol-3 phosphate pathway inducing changes in glycolysis via *GPD1L*. However, the results of the present study can only be used to suggest a genetic hypothesis; thus, further follow-up studies are needed.

Analysis of the enriched biological processes identified via GO analysis of the cohorts revealed that alpha-ketoglutarate-dependent dioxygenase FTO is related to sarcopenia. This finding is consistent with those of a previous study on the influences of *FTO* and muscle phenotypes^27^. In addition, alpha-ketoglutarate is a component of the tricarboxylic acid cycle, which is related to the HIF-1α pathway. This evidence suggests that a simultaneous understanding of both genes and gene-metabolic pathways is necessary to understand the pathogenesis of sarcopenia.

One of the primary strengths of this study is the utilisation of a relatively elderly cohort sample, which provides a better sarcopenic phenotype. Here, *NUDT3* and *RPS10* were replicated using a real cohort, which was an approach suggested by a previous study using the TWAS of muscle tissue^24^. Furthermore, our study focused on East Asian subjects, which have not been fully evaluated, unlike other ethnic groups. In this regard, we conducted phenome-wide association studies (pheWAS) using the “Common metabolic disease knowledge portal” (https://hugeamp.org), indicating that SNPs such as rs1187118, rs3768582, and rs6772958 are related to metabolic conditions such as waist-hip ratio, lipid metabolism, and body fat percentage in the European population (Supplementary Table S3).

Nevertheless, certain limitations were noted in this study. First, although novel signals for LBM and ASM were discovered with genome-wide significance, our results were based on bioinformatics analysis and, therefore, must be replicated in other Asian cohorts or multi-ethnic samples. A large number of samples for phenotypes, such as ASM and SMI, will improve the study’s validity. Hence, further studies, including replication or meta-analysis, are needed in other cohorts of the Asian population. Moreover, the number of SNPs (2,804,834) in the VHSMC cohort was limited as we set the imputation accuracy to 0.9. These points should be considered in the interpretation of the results. Second, the difference in ageing biology between sexes further hinders the identification of meaningful biomarkers for age-related conditions. Although GWAS analysis was conducted according to sex, the results did not show significant loci with genome-wide significance. It is expected that a metabolite-GWAS, considering sex as a factor, could help address this problem. Third, bioinformatics analysis revealed that genetic variants and metabolic pathways were related to sarcopenia, however, the causality of this hypothesis requires further investigation. Moreover, previous studies on genetic variants in sarcopenia have shown that these variants may be associated with the effects of genetic, metabolic, and environmental factors^22,27,28^. Fourth, we used bioelectrical impedance analysis (BIA) for LBM and BFM examinations, as it is a non-invasive method for measuring body composition. However, dual-energy X-ray absorptiometry (DXA) is the standard method for muscle mass. BIA and DXA have different limitations for studies using body composition measurements. A previous study that compared these two methods found that BIA overestimated ASM compared to DXA^41^. In addition, BIA devices differed in the two cohorts (InBody 3.0 for KARE cohort, InBody770 for VHSMC cohort), which may be a confounding factor. In a technical review of BIA for people with high body fat, InBody 3.0 tended to be lower, with a difference of about 2% in an extreme case (unpublished data). A previous study showed that different BIA devices were reliable by high intraclass correlation coefficients and low standard errors^42^. Since the focus of our study was on muscle mass rather than fat mass, and we analysed each cohort using different PCs, differences associated with the BIA device between the two cohorts would not significantly influence the LBM values and analysis results presented in this study. However, it is necessary to consider these when interpreting research results.

In conclusion, sarcopenia can result in adverse outcomes, such as an increased risk of falls, a decreased quality of life, and mortality. Thus, it is necessary to identify a biomarker for this condition. Here, the loci near genes such as *RPS10*, *NCF2*, *SMG7*, *ARPC5,* and *NUDT3* were identified to be significant biomarkers for LBM. In addition, the loci near *GPD1L* were identified as significant biomarkers for ASM and SMI, which serve as novel index for sarcopenia. These genes are related to metabolism pathways, such as glycerophospholipid pathways, energy metabolic pathway, the inositol phosphate and HIF-1α pathways, and alpha-ketoglutarate-dependent dioxygenase FTO. Further studies are required to evaluate the aetiology of sarcopenia.

## Methods

### Study subjects

Schematic plots of the analytical study design are shown in Fig. 1. Data were obtained from two cohorts: the VHSMC (n = 2518) and KARE (Ansan/Ansung study: from Korean Genome and Epidemiology Cohort, n = 5235) cohorts. Each cohort has its own distinct characteristics. The VHSMC cohort is a hospital-based elderly cohort that includes many patients with various diseases. The KARE cohort is a nationwide representative cohort for genome research in Korea; it is a longitudinal cohort of the Ansan and Ansung communities in Korea. This study included subjects from the KARE cohort and VHSMC cohort consisting of micro array data. Patients who had functional declines or limitations, or who had chronic diseases that may affect primary sarcopenia according to AWGS 2019^11^, were excluded. After exclusion, 6961 participants were enrolled across both cohorts (Fig. 1). The institutional review boards of the Veterans Health Service Medical Center approved this study protocol and informed consent waiver (IRB No. 2020-02-015 and IRB No. 2021-05-005), since this study was performed in a retrospective manner, and the study was conducted in compliance with the Helsinki Declaration. The committee of VHS Biobank (VBP-2020-03) and the National Biobank of Korea (KBN-2021-041) approved the use of bioresources for this study.

### Muscle mass measurement

BIA measurements were performed using InBody 770 (Biospace Co., LTD, Seoul, Korea) in the VHSMC cohort and using InBody 3.0 (Biospace Co., LTD, Seoul, Korea) in the KARE cohort. Each subject stood on the footplate and held both of the hand electrodes. The screen automatically displayed measurements of LBM (kg), skeletal muscle mass (kg), BFM (kg), and body fat percentage (%). LBM and BFM data were available for both cohorts and were used as initial phenotypes for analysis. Subgroup analysis was conducted using ASM or SMI, which were derived from BIA; these data were available only for the VHSMC cohort. The parameters were defined according to the consensus of the AWGS 2019^11^.

### Genotyping and imputation

Genomic DNA was separated from venous blood samples, and 100 ng DNA was genotyped using Korea Biobank Array Affymetrix Axiom 1.1 (Affymetrix, Santa Clara, CA), which was designed by the Korean National Institute of Health^43^. Genotypes were identified with a K-medoid clustering-based algorithm to minimise the batch effect^44^. The PLINK (version 1.9, Boston, MA)^45^ and ONETOOL^46^ software packages were used for quality control procedures and association analyses. Samples matching any of the following criteria were excluded: (1) sex inconsistencies or (2) a call rate of up to 97%. SNPs were filtered if the call rate was lower than the Hardy–Weinberg equilibrium (HWE) test (*P* < 1 ×  ${10}^{-5}$). The genotype imputation was conducted using the Michigan imputation server (https://imputationserver.sph.umich.edu). Only ‘non-European’ or ‘mixed’ populations from Haplotype Reference Consortium release v1.1^47^ were used for reference purposes. Pre-phasing and imputation were performed using Eagle v2.4^48^ and Minimac4^49^, respectively. After the imputation processes, imputed SNPs were removed if the R-squared (i.e., imputation accuracy) was less than 0.9 or there were duplicated SNPs, missing genotype rates were more extensive than 0.05, *P*-values for HWE were less than 1 ×  ${10}^{-5}$, or minor allele frequencies (MAFs) were less than 0.05. The MAF was compared with a reference such as Korean reference data (Kref) (http://coda.nih.go.kr) or the Genome Aggregation Database (GnomAD) with East Asian subjects (https://gnomad.broadinstitute.org/). Finally, 2422 subjects (and their 2,804,834 SNPs) from the VHSMC cohort and 5235 subjects (and their 3,423,819 SNPs) from the KARE cohort were used for analysis.

### Statistical analyses

Baseline characteristics of the study population are presented herein as means with standard deviation (SD) for continuous variables and numbers, and as proportions for categorical variables. Genome-wide analyses were conducted using a linear model; PLINK was used within each cohort. Age, sex, and ten principal component scores were included as covariates. Meta-analyses of the VHSMC and KARE cohorts were performed using the METAL software (http://csg.sph.umich.edu/abecasis/meta). Cochran’s Q-test for heterogeneity was conducted; its *P*-value was marked with ‘*HetPVal’*^50^, where *HetPVal* < 0.05 indicates heterogeneity between two datasets^51^. The dense regional association result of each GWAS was plotted using the LocusZoom software^52^. The threshold for statistical significance in this model was *P* < 5.0 ×  ${10}^{-8}$, which is conventionally considered to reflect genome-wide significance.

### Functional annotation analyses

Expression Quantitative trait (eQTL) studies were performed using the Genotype-Tissue Expression (GTEx) dataset (https://gtexportal.org/home/), which provides a variety of human tissues from donors using the densely genotyped data to assess genetic variations within their genomes. Genes related to metabolites were analysed using KEGG pathway analysis^31^. Associated genes were further investigated for DEGs in the skeletal muscles of subjects 19 to 28 and 65 to 76 years of age from the Gene Expression Omnibus (GEO) dataset (GSE38718)^53^. In addition, biological process, cellular component, and molecular function GO analyses were performed using gene set enrichment analysis. The Benjamini–Hochberg false discovery rate (FDR)-adjusted 0.1 significance level was applied for multiple hypothesis test corrections^54^.

### Ethics declarations

The institutional review boards of the Veterans Health Service Medical Center approved this study protocol and informed consent waiver (IRB No. 2020-02-015 for *VHSMC cohort* and IRB No. 2021-05-005 for *KARE cohort*) since this study was performed in retrospective manner, and the study was conducted in compliance with the Helsinki Declaration.

### Consent to participate

Informed consent waiver was approved by the institutional review boards of the Veterans Health Service Medical Center since this study was performed in a retrospective manner and anonymised and de-identified data were used for the analyses. The KARE cohort and VHSMC cohort obtained the informed consents from participants.

## Supplementary Information

Supplementary Information.

## Data Availability

The data supporting the findings of this study are available upon reasonable request.

## References

[1] Shafiee G (2017). Prevalence of sarcopenia in the world: A systematic review and meta-analysis of general population studies. J. Diabetes Metab. Disord..

[2] Tanimoto Y (2012). Association between sarcopenia and higher-level functional capacity in daily living in community-dwelling elderly subjects in Japan. Arch. Gerontol. Geriatr..

[3] Cesari M (2009). Skeletal muscle and mortality results from the InCHIANTI study. J. Gerontol. A Biol. Sci. Med. Sci..

[4] Janssen I, Shepard DS, Katzmarzyk PT, Roubenoff R (2004). The healthcare costs of sarcopenia in the United States. J. Am. Geriatr. Soc..

[5] McNamee P, Bond J, Buck D, Ageing S, Resource Implications Study of the Medical Research Council Cognitive, F. (2001). Costs of dementia in England and Wales in the 21st century. Br. J. Psychiatry.

[6] Chen LK (2016). Recent advances in sarcopenia research in Asia: 2016 update from the Asian Working Group for Sarcopenia. J. Am. Med. Dir. Assoc..

[7] Chen LK (2014). Sarcopenia in Asia: Consensus report of the Asian Working Group for Sarcopenia. J. Am. Med. Dir. Assoc..

[8] Morley JE (2011). Sarcopenia with limited mobility: An international consensus. J. Am. Med. Dir. Assoc..

[9] Fielding RA (2011). Sarcopenia: An undiagnosed condition in older adults. Current consensus definition: Prevalence, etiology, and consequence. International working group on sarcopenia. J. Am. Med. Dir. Assoc..

[10] Cruz-Jentoft AJ (2010). Sarcopenia: European consensus on definition and diagnosis: Report of the European Working Group on Sarcopenia in Older People. Age Ageing.

[11] Chen LK (2020). Asian Working Group for Sarcopenia: 2019 consensus update on sarcopenia diagnosis and treatment. J. Am. Med. Dir. Assoc..

[12] Roubenoff R (2003). Sarcopenia: Effects on body composition and function. J. Gerontol. A Biol. Sci. Med. Sci..

[13] Rolland Y (2008). Sarcopenia: Its assessment, etiology, pathogenesis, consequences and future perspectives. J. Nutr. Health Aging.

[14] Arden NK, Spector TD (1997). Genetic influences on muscle strength, lean body mass, and bone mineral density: A twin study. J. Bone Miner. Res..

[15] Liu XG (2009). Genome-wide association and replication studies identified TRHR as an important gene for lean body mass. Am. J. Hum. Genet..

[16] Hai R (2012). Genome-wide association study of copy number variation identified gremlin1 as a candidate gene for lean body mass. J. Hum. Genet..

[17] Guo YF (2013). Suggestion of GLYAT gene underlying variation of bone size and body lean mass as revealed by a bivariate genome-wide association study. Hum. Genet..

[18] Urano T, Shiraki M, Sasaki N, Ouchi Y, Inoue S (2014). Large-scale analysis reveals a functional single-nucleotide polymorphism in the 5′-flanking region of PRDM16 gene associated with lean body mass. Aging Cell.

[19] Ran S (2014). Genome-wide association study identified copy number variants important for appendicular lean mass. PLoS ONE.

[20] Trajanoska K, Rivadeneira F, Kiel DP, Karasik D (2019). Genetics of bone and muscle interactions in humans. Curr. Osteoporos. Rep..

[21] Urano T, Inoue S (2015). Recent genetic discoveries in osteoporosis, sarcopenia and obesity. Endocr. J..

[22] Zillikens MC (2017). Large meta-analysis of genome-wide association studies identifies five loci for lean body mass. Nat. Commun..

[23] Han Z (2010). Crystal structure of the FTO protein reveals basis for its substrate specificity. Nature.

[24] Singh AN, Gasman B (2020). Disentangling the genetics of sarcopenia: Prioritization of NUDT3 and KLF5 as genes for lean mass & HLA-DQB1-AS1 for hand grip strength with the associated enhancing SNPs & a scoring system. BMC Med. Genet..

[25] Khanal P (2020). Prevalence and association of single nucleotide polymorphisms with sarcopenia in older women depends on definition. Sci. Rep..

[26] Jones G (2020). Sarcopenia and variation in the human leukocyte antigen complex. J. Gerontol. A Biol. Sci. Med. Sci..

[27] Heffernan SM (2017). Fat mass and obesity associated (FTO) gene influences skeletal muscle phenotypes in non-resistance trained males and elite rugby playing position. BMC Genet..

[28] Hebbar P (2019). FTO variant rs1421085 associates with increased body weight, soft lean mass, and total body water through interaction with ghrelin and apolipoproteins in Arab population. Front. Genet..

[29] Jones G (2021). Genome-wide meta-analysis of muscle weakness identifies 15 susceptibility loci in older men and women. Nat. Commun..

[30] Arce-Cerezo A (2015). HMGA1 overexpression in adipose tissue impairs adipogenesis and prevents diet-induced obesity and insulin resistance. Sci. Rep..

[31] Kanehisa M, Furumichi M, Sato Y, Ishiguro-Watanabe M, Tanabe M (2021). KEGG: Integrating viruses and cellular organisms. Nucleic Acids Res..

[32] Narasimhan A, Greiner R, Bathe OF, Baracos V, Damaraju S (2018). Differentially expressed alternatively spliced genes in skeletal muscle from cancer patients with cachexia. J. Cachexia Sarcopenia Muscle.

[33] Fochi S (2020). Regulation of microRNAs in satellite cell renewal, muscle function, sarcopenia and the role of exercise. Int. J. Mol. Sci..

[34] Bolotta A (2020). Skeletal muscle gene expression in long-term endurance and resistance trained elderly. Int. J. Mol. Sci..

[35] Chaves DF (2013). Comparative proteomic analysis of the aging soleus and extensor digitorum longus rat muscles using TMT labeling and mass spectrometry. J. Proteome Res..

[36] Gueugneau M (2014). Proteomics of muscle chronological ageing in post-menopausal women. BMC Genomics.

[37] He H (2017). A systems genetics approach identified GPD1L and its molecular mechanism for obesity in human adipose tissue. Sci. Rep..

[38] He N (2020). Circulating microRNAs in plasma decrease in response to sarcopenia in the elderly. Front. Genet..

[39] Moaddel R (2016). Plasma biomarkers of poor muscle quality in older men and women from the Baltimore longitudinal study of aging. J. Gerontol. A Biol. Sci. Med. Sci..

[40] Gonzalez-Freire M (2019). Targeted metabolomics shows low plasma lysophosphatidylcholine 18:2 predicts greater decline of gait speed in older adults: The Baltimore longitudinal study of aging. J. Gerontol. A Biol. Sci. Med. Sci..

[41] Lee SY (2018). Comparison between dual-energy X-ray absorptiometry and bioelectrical impedance analyses for accuracy in measuring whole body muscle mass and appendicular skeletal muscle mass. Nutrients.

[42] McLester CN, Nickerson BS, Kliszczewicz BM, McLester JR (2020). Reliability and agreement of various inbody body composition analyzers as compared to dual-energy X-ray absorptiometry in healthy men and women. J. Clin. Densitom.

[43] Moon S (2019). The Korea biobank array: Design and identification of coding variants associated with blood biochemical traits. Sci. Rep..

[44] Seo S (2019). SNP genotype calling and quality control for multi-batch-based studies. Genes Genomics.

[45] Purcell S (2007). PLINK: A tool set for whole-genome association and population-based linkage analyses. Am. J. Hum. Genet..

[46] Song YE (2018). ONETOOL for the analysis of family-based big data. Bioinformatics.

[47] McCarthy S (2016). A reference panel of 64,976 haplotypes for genotype imputation. Nat. Genet..

[48] Loh P-R (2016). Reference-based phasing using the Haplotype Reference Consortium panel. Nat. Genet..

[49] Das S (2016). Next-generation genotype imputation service and methods. Nat. Genet..

[50] Cochran WG (1954). The combination of estimates from different experiments. Biometrics.

[51] Ioannidis JP (2008). Interpretation of tests of heterogeneity and bias in meta-analysis. J. Eval. Clin. Pract..

[52] Pruim RJ (2010). LocusZoom: Regional visualization of genome-wide association scan results. Bioinformatics.

[53] Raue U (2012). Transcriptome signature of resistance exercise adaptations: Mixed muscle and fiber type specific profiles in young and old adults. J. Appl. Physiol..

[54] Benjamini Y, Hochberg Y (1995). Controlling the false discovery rate: A practical and powerful approach to multiple testing. J. R. Stat. Soc. Ser. B (Methodol.).

