# Supplementary material for: Unveiling genetic variants for age-related sarcopenia by conducting a genome-wide association study on Korean cohorts
Source: Sci Rep. 2022 Mar 3;12:3501. doi: 10.1038/s41598-022-07567-9 (PMC8894365; doi:10.1038/s41598-022-07567-9)
Supplement: Supplementary file 1 — Supplementary Information. [file 41598_2022_7567_MOESM1_ESM.docx]

**
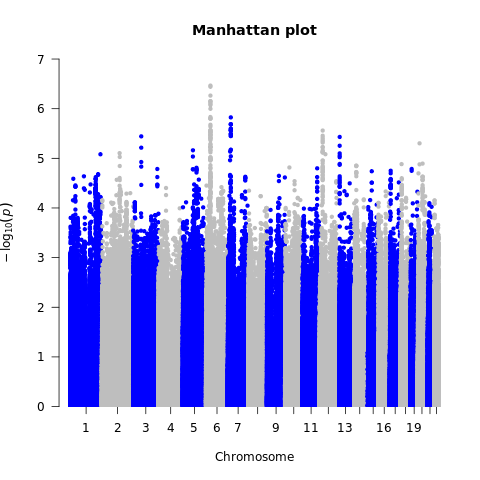
(A)**

**
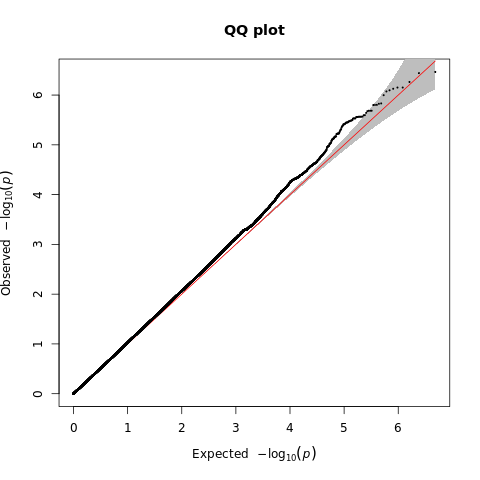
(B)**

**Supplementary Figure S1. Manhattan and quantile-quantile plot for body fat mass in meta-analysis. (A)** Manhattan plot of the *P*-values in the genome-wide association study (GWAS) meta-analysis for body fat mass. **(B)** Quantile-quantile (Q-Q) plot showing expected vs. observed [−log_10_(*P*)values]. The expected line is shown in red and confidence bands are shown in grey.

**
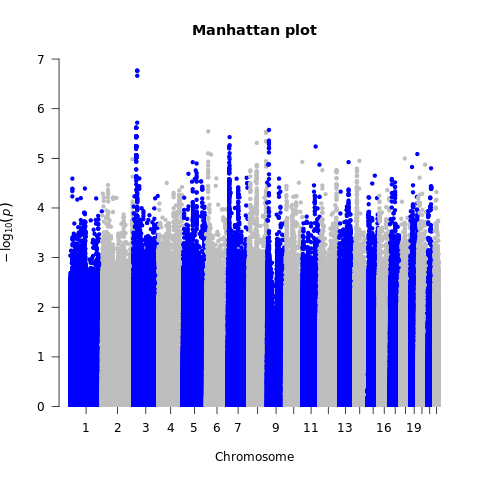
 (A)**

**
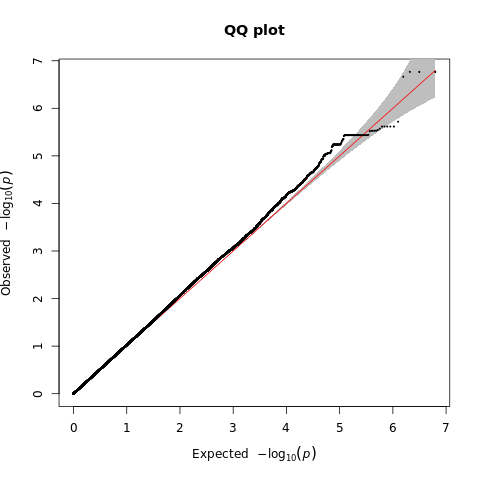
**

**(B)**

**Supplementary Figure S2. Manhattan and quantile-quantile plot for skeletal muscle index in genome-wide association analysis. (A)** Manhattan plot of the *P*-values in the GWAS meta-analysis for skeletal muscle index. **(B)** Q-Q plot showing expected vs. observed [−log_10_(*P*)values]. The expected line is shown in red and confidence bands are shown in grey.

**Supplementary Table S1. Results of GWAS meta-analysis for body fat mass (top ten leading SNPs).**

|  |  |  |  |  | **Independent study** | | | | | **Meta-analysis** | | |  |
| --- | --- | --- | --- | --- | --- | --- | --- | --- | --- | --- | --- | --- | --- |
| **Chr** | **SNP** | **Locus** | **A** | **MAF** | **Cohort** | **Effect** | **SE** | **MAF** | ***P*** | **Effect** | **SE** | ***P***  ***(*HetPVal)** | **Mapped Genes** |
| 6 | rs1592269 | 34183057 | G/A | 0.16^a^, 0.10^b^ | VHSMC | 1.054 | 0.293 | 0.16 | $3.42\times{10}^{-4}$ | 0.753 | 0.148 | $3.43\times{10}^{-7}$  (0.3795) | *GRM4, HMGA1* (intergenic) |
|  |  |  |  |  | KARE | 0.649 | 0.173 | 0.16 | $1.88\times{10}^{-4}$ |  |  |  |  |
| 7 | rs73085119 | 22520389 | C/T | 0.08^a^, 0.10^b^ | VHSMC | -0.056 | 0.263 | 0.09 | $6.73\times{10}^{-4}$ | 0.425 | 0.175 | $2.07\times{10}^{-6}$  (0.3693) | *STEAP1B*  (intronic) |
|  |  |  |  |  | KARE | 0.810 | 0.235 | 0.08 | $5.99\times{10}^{-4}$ |  |  |  |  |
| 12 | rs10842139 | 23314305 | G/A | 0.08^a^, 0.10^b^ | VHSMC | 1.284 | 0.413 | 0.08 | $1.90\times{10}^{-3}$ | 0.943 | 0.203 | $2.76\times{10}^{-6}$  (0.5614) | *ETNK1, LOC101928441* (intergenic) |
|  |  |  |  |  | KARE | 0.834 | 0.234 | 0.08 | $3.69\times{10}^{-4}$ |  |  |  |  |
| 3 | rs4770186 | 63352139 | T/C | 0.17^a^, 0.18^b^ | VHSMC | -0.976 | 0.283 | 0.16 | $5.89\times{10}^{-4}$ | -0.682 | 0.148 | $3.61\times{10}^{-6}$  (0.3083) | *SYNPR* (intronic) |
|  |  |  |  |  | KARE | -0.572 | 0.174 | 0.16 | $1.07\times{10}^{-3}$ |  |  |  |  |
| 13 | rs4770186 | 22226375 | C/T | 0.37^a^, 0.46^b^ | VHSMC | 0.389 | 0.225 | 0.35 | $8.48\times{10}^{-2}$ | 0.538 | 0.114 | $3.71\times{10}^{-6}$  (0.2964) | MICU2, FGF9 (intergenic) |
|  |  |  |  |  | KARE | 0.591 | 0.133 | 0.36 | $9.90\times{10}^{-6}$ |  |  |  |  |
| 20 | rs6134616 | 12640010 | T/C | 0.06^a^, 0,06^b^ | VHSMC | -1.363 | 0.450 | 0.06 | $2.52\times{10}^{-3}$ | -1.074 | 0.236 | $4.98\times{10}^{-6}$  (0.5738) | *BTBD3, LINC01722*  (intergenic) |
|  |  |  |  |  | KARE | -0.965 | 0.278 | 0.06 | $5.21\times{10}^{-4}$ |  |  |  |  |
| 5 | rs337892 | 78222966 | C/T | 0.26^a^, 0.33^b^ | VHSMC | -0.528 | 0.241 | 0.26 | $2.87\times{10}^{-2}$ | -0.565 | 0.124 | $6.89\times{10}^{-6}$  (0.6906) | *ARSB* (intronic) |
|  |  |  |  |  | KARE | -0.579 | 0.146 | 0.26 | $7.81\times{10}^{-5}$ |  |  |  |  |
| 7 | rs6979038 | 16927752 | A/G | 0.44^a^, 0.45^b^ | VHSMC | -0.616 | 0.217 | 0.44 | $4.61\times{10}^{-3}$ | -0.496 | 0.110 | $7.30\times{10}^{-6}$  (0.6960) | *AGR3, AHR* (intergenic) |
|  |  |  |  |  | KARE | -0.454 | 0.129 | 0.44 | $4.68\times{10}^{-4}$ |  |  |  |  |
| 12 | rs7969016 | 44309726 | C/G | 0.18^a^, 0.24^b^ | VHSMC | 0.636 | 0.313 | 0.14 | $4.25\times{10}^{-2}$ | 0.648 | 0.143 | $8.27\times{10}^{-6}$  (0.5740) | *TMEM117* (intronic) |
|  |  |  |  |  | KARE | 0.652 | 0.162 | 0.20 | $6.08\times{10}^{-5}$ |  |  |  |  |
| 1 | rs2275400 | 236992753 | A/C | 0.09^a^, 0.09^b^ | VHSMC | -1.142 | 0.389 | 0.09 | $3.38\times{10}^{-3}$ | -0.858 | 0.194 | $8.29\times{10}^{-6}$  (0.5986) | *MTR* (intronic) |
|  |  |  |  |  | KARE | -0.764 | 0.224 | 0.09 | $6.73\times{10}^{-4}$ |  |  |  |  |

Chr, chromosome; SNP, single nucleotide polymorphism; MAF, minor allele frequency; SE, standard error; Mapped Genes from ANNOVAR; GWAS, genome-wide association study; VHSMC, Veterans Health Service Medical Center; KARE, Korean Association Resource

**^a^**Kref, Korean reference data; **^b^**GnomAD Genome Aggregation Database (East Asian)

**Supplementary Table S2. Results of GWAS for skeletal muscle index (top ten leading SNPs).**

| **Chr** | **SNP** | **Position** | **Allele** | **MAF** | **Effect** | **SE** | ***P*** | **Mapped Genes** |
| --- | --- | --- | --- | --- | --- | --- | --- | --- |
| 3 | rs6772958 | 32049091 | A/G | 0.39^a^, 0.39^b^, 0.45^c^ | -0.121 | 0.023 | $1.72\times{10}^{-7}$ | *ZNF860, GPD1L* (intergenic) |
| 9 | rs527705 | 15201720 | A/C | 0.30 ^a^, 0.31^b^, 0.33^c^ | 0.116 | 0.024 | $2.69\times{10}^{-6}$ | *TTC39B* (intronic) |
| 6 | rs2146753 | 16694501 | A/G | 0.34 ^a^, N/A, 0.34^c^ | -0.113 | 0.002 | $2.87\times{10}^{-6}$ | *ATXN1* (intronic) |
| 8 | rs11166746 | 138701808 | A/T | 0.46 ^a^, 0.46^b^, 0.48^c^ | 0.107 | 0.022 | $2.97\times{10}^{-6}$ | *LOC101927915, LOC401478* (intergenic) |
| 3 | rs183170893 | 23917412 | C/T | 0.05 ^a^, 0.05^b^, 0.05^c^ | 0.239 | 0.051 | $3.61\times{10}^{-6}$ | *UBE2E1* (intronic) |
| 7 | rs2192326 | 13295115 | A/G | 0.18 ^a^, 0.18^b^, 0.18^c^ | 0.134 | 0.028 | $3.74\times{10}^{-6}$ | *ARL4A, ETV1*(intergenic) |
| 11 | rs145598934 | 102996084 | T/C | 0.05 ^a^, 0.03^b^, 0.03^c^ | 0.223 | 0.049 | $5.78\times{10}^{-6}$ | *DYNC2H1* (intronic) |
| 19 | rs62143162 | 54298374 | G/C | 0.19 ^a^, N/A, 0.16^c^ | -0.128 | 0.028 | $8.20\times{10}^{-6}$ | *NLRP12* (intronic) |
| 6 | rs2003132 | 39031989 | C/A | 0.44 ^a^, 0.43^b^, 0.49^c^ | 0.104 | 0.023 | $8.39\times{10}^{-6}$ | *GLP1R* (intronic) |
| 3 | rs72628116 | 24027540 | C/G | 0.05 ^a^, 0.05^b^, 0.06^c^ | 0.233 | 0.052 | $9.78\times{10}^{-6}$ | NR1D2, LINC00691 (intergenic) |

Chr, chromosome; SNP, single nucleotide polymorphism; MAF, minor allele frequency; SE, standard error; Mapped Genes from ANNOVAR; GWAS, genome-wide association study

**^a^**VHSMC, Veterans Health Service Medical Center; **^b^**Kref, Korean reference data; **^c^**GnomAD, Genome Aggregation Database (East Asian)

**Supplementary Table S3. Links to Phenome-wide association study (pheWAS) using “Common Metabolic Diseases Knowledge Portal” (**[**https://hugeamp.org/**](https://hugeamp.org/)**).**

PheWAS result for significant SNP (rs1187118) in lean body mass (<https://hugeamp.org/variant.html?variant=rs1187118>)

PheWAS result for significant SNP (rs3768582) in lean body mass (<https://hugeamp.org/variant.html?variant=rs3768582>)

PheWAS result for significant SNP (rs6772958) in appendicular skeletal muscle mass (<https://hugeamp.org/variant.html?variant=rs6772958>)
